# Psychological coping in female breast cancer patients from a Metropolitan Lima hospital

**DOI:** 10.17843/rpmesp.2022.394.12322

**Published:** 2022-12-22

**Authors:** Fiorella D. Rivera-Cruzatt, Pierina P. Cubillas-Espinoza, Eli L. Malvaceda-Espinoza

**Affiliations:** 1 Universidad San Ignacio de Loyola, Lima, Peru. Universidad San Ignacio de Loyola Universidad San Ignacio de Loyola Lima Peru

**Keywords:** Coping Strategies, Patient, Women, Breast Neoplasms, Qualitative Research

## Abstract

**Objective.:**

To understand the psychological coping strategies in female patients with breast cancer from a hospital in Metropolitan Lima.

**Materials and methods.:**

This was a qualitative research with a reflexive thematic analysis design. Sixteen women with breast cancer between 35 and 65 years of age were interviewed. Data was analyzed with the ATLAS.ti 22 software.

**Results.:**

Three psychological coping strategies were described: emotional coping, which was found more frequently, includes the support of important people, religious coping, and focusing on positive consequences, which leads to a positive reinterpretation and progressive acceptance of the disease; active coping, characterized by diligent action, following indications, and seeking professional support. Finally, avoidance coping, which focuses on negative elements, postponement of the coping process and cognitive and behavioral distractions, the latter being of utmost relevance to balance the activities in the patients’ daily lives.

**Conclusions.:**

Participants used emotional coping strategies more frequently, since they tried to increase positive emotions, accompanied by religious and environmental support. In addition, they also used active coping strategies, since they focused their actions to receive medical attention and treatment, leaving aside other activities; in spite of this, they used strategies to take their focus off the condition and thus detach themselves from their worries.

## INTRODUCTION

In 2020, breast cancer caused the death of approximately 685,000 women worldwide and was detected in about 2.2 million women ^1^. During that year, 6860 cases were diagnosed in Peru, of which 63% were between 35 and 64 years of age ^2^^,^^3^. Treatment for this condition is affected by the health status, eating disorders, consumption behavior and oral contraceptive methods ^4^. Thus, medical treatment can range from surgery to general removal of breast tissue, combined with chemotherapy, radiotherapy, pharmacological therapy, among others; these treatments can trigger side effects both in the reproductive and sexual aspect, such as the inability to have children, which creates greater concern in women ^5^^,^^6^.

Likewise, women face states of vulnerability, confusion, worry, hopelessness and uncertainty during while adapting to the diagnosis, ^7^^-^^10^. This causes low self-esteem ^11^, feelings of inferiority ^7^, emotions like anger, guilt, grief and fear ^7^^,^^8^^,^^12^^,^^13^, and can even trigger disorders such as depression and anxiety ^7^^,^^14^, as well as high levels of stress ^7^^,^^9^^,^^10^. These psychological effects increase in a pandemic context, since it is more difficult to obtain essential elements by maintaining social distancing, besides women prefer to maintain communication with online support groups because they perceive limited social support from friends and family ^4^^,^^15^.

Even when family support is provided, economic problems and communication failures can cause the separation of some family members, resulting in feelings of frustration and misunderstandings, even when support is positive, physical or emotional dependence can occur ^12^^,^^13^. Therefore, it is essential to understand the coping strategies that women use during and after learning of their diagnosis ^7^^,^^14^. In this sense, psychological coping is understood as the cognitive or behavioral tasks carried out by the person, in order to psychosocially adapt to a problem ^16^. Such coping is divided into strategies, based on a group of actions that vary according to the person’s environment and other factors ^17^.

In this regard, the emotional coping style ^16^^,^^18^ includes strategies (family and friend support, religious support, positive transformation, denial and acceptance) that allow adjusting the level of emotional suffering caused by such health condition ^19^. In addition, the resolute coping style ^16^^,^^18^, involves strategies (diligence, professional support, postponement of coping actions, planning, omission of ordinary activities), with the aim of reducing the stress, and promoting positive psychological and emotional attitudes ^17^^,^^18^^,^^20^. The avoidant coping style ^18^ includes strategies (concentration and manifestation of adverse effects, cognitive distraction and behavioral distraction), where the individual shows disinterest in the intimidating event ^18^^,^^19^ in order to avoid the problem.

Previous research shows that, globally, the most used coping strategies by women are related to conflict solving ^17^^,^^20^. In addition, variables such as age, sentimental status and schooling are taken into account when choosing a strategy ^21^. In Peru, the emotional coping style is the most used one and it involves religious coping and positive reinterpretation strategies ^19^^,^^22^; however, avoidant coping strategies are less frequent ^22^. In this regard, patients show that the more professional support they receive, the greater their psychological coping will be ^7^; likewise, this support can come from the healthcare personnel, by being warm and empathetic, encouraging healthy eating habits, hygiene and showing a positive emotional state ^23^.

These studies were conducted in Peru and followed a quantitative approach, so they did not collect in-depth information on the phenomenon; therefore, we aim to address this theoretical gap through empirical evidence using qualitative methodological tools. At the same time, this will help more women to identify and use positive coping strategies, besides showing the importance of the psychological support provided to them. Accordingly, this study aims to understand psychological coping of female patients with breast cancer in a hospital in Metropolitan Lima.

**KEY MESSAGES**

Motivation for the study: it is important to identify the coping strategies used by women with breast cancer, in order to contribute to their physical and emotional well-being.

Main findings: strategies related to the emotional sphere are used to a greater extent, and also lead to progressive acceptance of the disease. Cognitive and behavioral distractions are necessary to balance patients’ daily activities.

Implications: understanding how women face this disease allows the development of primary care strategies to improve their wellbeing.

## MATERIALS AND METHODS

### Type and design

This study followed a qualitative approach and had a reflexive thematic analysis design. The former allowed for in-depth analysis of the study subject in its natural environment, reviewing the data and determining its categories ^24^. The design of this research allows the understanding of the information by focusing on the topics determined by the participants and explained by the researchers, associating the data according to patterns in relevant topics ^25^.

### Participants

We included sixteen women aged 35 to 65 years (Table 1) diagnosed with breast cancer who received medical care at a level-3 public hospital in Metropolitan Lima, which specializes in oncological care. We applied convenience sampling, which is used when there is little accessibility to the population ^26^; this is the case of the present study, where the subject matter is associated with severe disease.

**Table 1:** Characteristics of the participants.

Pseudonym	Age	Employment	Marital status	Family composition	Stage at diagnosis	Age at diagnosis
AA	64	Nurse	Married	Husband and son	II	63
OS	46	RH	Married	Husband and sons	II	44
DA	59	RH	Married	Husband and son	III	54
DN	52	Nursing Technician	Married	Husband and sons	II	51
JI	59	Independent	Married	Children and niece	II	58
TC	56	Administrator	Married	Husband and sons	II	55
MR	48	Teacher	Married	Husband and sons	II	47
AR	65	RH	Single	Sister and niece	III	60
SU	51	Independent	Single	Mother and siblings	III	49
YJ	59	RH	Married	Husband and sons	III	56
LG	64	Independent	Married	Husband	III	63
TH	59	RH	Married	Husband and daughters	III	58
SF	51	Cosmetologist	Married	Husband	II	48
RS	48	Independiente	Married	Son, parents and siblings	III	43
SN	35	Accountant	Single	Father	III	31
CR	51	RH	Married	Husband and sons	II	46

RH: responsible for the household, II: stage 2 breast cancer, III: stage 3 breast cancer.

Patients in stages II and III were included, detected at least one year before this research. Those who presented physical comorbidity or some psychopathological alterations were excluded, due to the differences in terms of treatment, physical and psychological consequences, process of adaptation to the diagnosis, professional and family support, as well as in the care provided by the hospitals that specialize in cancer ^5^^,^^13^.

### Instruments for data collection

We used a semi-structured interview (Table 2) that was constructed based on the theoretical contributions about psychological coping explained from the topics of emotional, resolute and avoidant coping ^18^. The interview guide was evaluated by ten expert judges, health professionals knowledgeable about cancer patients, who evaluated the clarity, coherence and representativeness of the questions; their approval was obtained in order to carry out the interviews. A pilot interview was conducted by telephone call, which helped refine the questions for the final interview guide.

**Table 2:** Guiding questions.

Topics	Guiding questions
Emotional coping	Who did you turn to for emotional support during your illness? Why seek such support from others?
Resolute coping	At what point did you begin to take action to address the illness? Do you consider that your actions were taken at the right time? Why?
Avoidant coping	What aspects of your illness do you consider that you focus on? How do you express the emotions derived from these consequences? Do you consider it appropriate?

Source: own elaboration.

### Procedures

First, we reached out to the president of a group of breast cancer patients from a hospital in Lima via a social network; with her approval, the patients were contacted for the first time. We contacted 37 women, 17 of whom agreed to participate in the research; however, only 16 met the inclusion criteria. Subsequently, the participants were informed about the purpose of the research. They gave their informed consent orally, which was recorded on audio files. Likewise, they were told about the confidentiality, anonymity (use of pseudonyms), beneficence and nonmaleficence principles that guided this research.

Subsequently, the interviews were scheduled and then conducted via telephone calls. During the telephone calls, we ensured that the participants were alone and in a comfortable space (usually the living room of their home). The interviews were conducted between September and October 2020, and lasted an average of 40 minutes. The interviews, as well as the verbatim transcription, were carried out by the researchers FR and PC, who worked on an equal number of interviews. No patient refused to participate or dropped out of the study. Likewise, no payment was given for participating in the interviews, which were conducted in one session (there were no repetitions) and in Spanish.

### Data analysis

We followed the guidelines of reflexive thematic analysis with a hybrid method ^25^. The process started with the familiarization with the data, through transcriptions. Sub sequently, initial codes were generated and assigned to the relevant quotations in the document; we applied a double cycle of axial and selective coding by patterns ^27^. In parallel, we generated, reviewed, and defined the themes, which were identified by the constant comparative method, grouping the codes into categories. Finally, we prepared the results report, where the strategies of meaning generation were found ^28^, such as the identification of patterns, which allows evidencing the saturation of the codes and/or categories; the count, which allows showing the magnitude of the codes throughout the analysis; and the density, which evidences the explanatory power of the codes, through their connections with other codes and/or categories. The categories that fulfilled at least one of the aforementioned tactics were considered as major themes. The analysis was carried out mainly by FR and PC, and they discussed and solved the discrepancies in the coding together with MS. The analysis was carried out with the support of ATLAS.ti 22 software.

### Ethical and quality criteria

Permission was obtained from the ethics committee of the Graduate School of the Universidad Peruana Unión (No. 2022-CE-EPG-0000116). This article is adapted to the criteria of the Consolidated Criteria for Reporting Qualitative Research (COREQ) ^29^.

The present study meets quality criteria such as credibility ^30^, since the transcripts and results of the study were given to the participants to verify the information and obtain their opinions; however, there were no additional comments from them. It also complies with the confirmability criterion ^30^, since data collection, analysis and results can be traced and verified to their origin, which generates a logical chain of evidence. Next, auditability ^31^, which implied the triangulation of researchers by auditing the interview guide through 10 expert judges.

## RESULTS

The 16 participants interviewed had an average age of 54 years. Stage II and III patients were distributed equally. Regarding their activities, six indicated that they were responsible for the household, while the other 10 had a trade or profession. Most participants were married and their family structure consisted of a spouse and children or one of them. The ages at which they were diagnosed ranged from 31 to 63 years.

According to the analysis, breast cancer causes different psychosocial consequences, including dependence, physical weakness, as well as feelings of sadness, depression and frustration. 

*There I was, with more pain in my body, my bones hurt, I could no longer walk as much and I was in a wheelchair. SN, 35 years old.*

**Figure 1:**
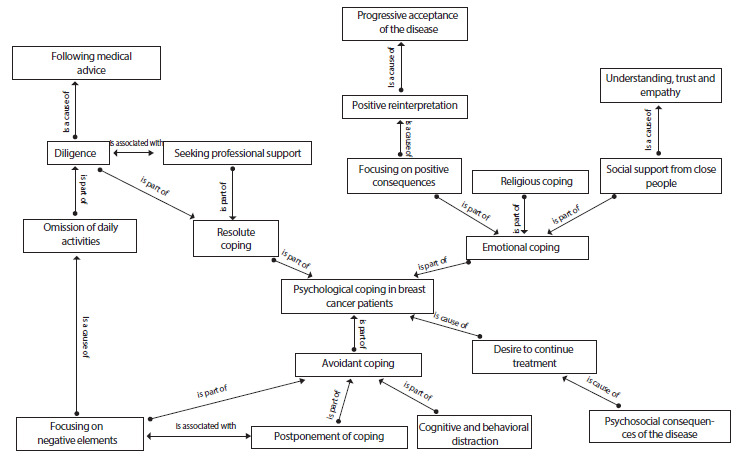
Semantic map of coping strategies in female breast cancer patients.

In spite of this, participants showed a desire to continue treatment. This leads the participants to cope psychologically with their illness through different strategies such as emotional, resolutive and avoidant coping, which will be discussed below (Figure 1).

### Emotional coping

This coping strategy was the most frequently found and involves actions focused on emotions, with the aim of improving the current situation. In this regard, the patients sought social support from close people, whether from family, friends, support groups or religious groups, this was important for stage III patients. 

*In my case, my friends at work told me: cheer up, SN, seek another opinion. SN, 35 years old.*

This is a direct way of coping, with the aim of seeking understanding, trust and empathy. They also showed religious coping, which helped them become closer and strengthened their beliefs.

*I am a believer, that was my support* [*...* ] *I believed that God’s will was being clear with the relapses and I had to accept it* [*...* ] *I am moving forward for my healing. CR, 51 years old.*

On the other hand, participants also showed that they focus on the positive consequences of the disease, which leads them to generate a positive reinterpretation, as they recognize beneficial experiences in the face of what they have lived; at this point, they point out to carry out new activities and revalue daily activities (spending time with the family and improving self-care).

*But as a result of this diagnosis, I have decided to do things that I was always thought of doing later. So, now I have dedicated myself to music, I plan to teach, it fills my soul, my life and everything. It’s something I enjoy. TC, 56 years old.*

An element that emerges from the positive reinterpretation is the progressive acceptance of the disease, thus, although they had feelings of fear and worry at the beginning, they later experienced surprise and tranquility, and came to accept the disease.

*One has to live with it, there is nothing else to do, although you still miss your life before, the things you used to do. JN, 58 years old.*

In this sense, women sought support from close personal environment, in search of understanding, trust and empathy, which made them feel accompanied in order to control the emotions caused by the disease. 

Likewise, approaching religion made them feel that the disease is an obstacle to overcome, which gave them faith and strength, making them feel comforted while they focused on the positive consequences, either to avoid increasing negative emotions or because they had a more optimistic perspective of life situations in accordance with their personality, so they could have a positive reinterpretation, achieving a progressive acceptance of the disease.

### Resolute coping

The patients, during their first experiences with the diagnosis, took diligent actions to intervene directly in the face of suspicions about their condition, especially when seeking a medical opinion to confirm their diagnosis and learn about the possible treatments they would have to undergo. In the face of this, they focused on following medical indications. 

*Just following what the doctors told me, right? They are the ones who tell you, first the surgery, the mastectomy, radiation, biopsy, an appointment for this, for that, they are the ones you have to follow. SF, 51 years old.*

Therefore, the patients sought professional support, both physical and psychological, in order to reduce the adverse effects of the disease, which is representative of women in stage II. 

*When I went to the psychologist, he helped me a lot because I felt that this person made me feel calmer, I felt the hugs from the doctor who supported me, he always tells me: You are getting over it, you are doing fine. DN, 52 years old.*

On the other hand, participants stated that they omitted daily activities, whether work, academic or home activities that they carried out before learning of their diagnosis, in order to focus all their temporal, mental and physical efforts to carry out the treatment and its implications.

*Before, everything was normal, but when I found out, since then, I have been almost a year without working, since the treatment, the operations, the chemotherapy and now the radiotherapy take too much time* [*...* ] *so, it is practically dedicating all that time to just cure yourself. TC, 56 years old.*

Thus, the suspicion of breast cancer produces uncertainty, so in order to reduce it, patients seek medical attention to learn about their condition. When they already know about it, they focus on complying with medical indications, due to hasty decision making to avoid aggravation of their health. Thus, they seek professional support to improve their physical and mental condition; likewise, they are committed to set aside routine activities, studies and work, due to lack of time, motivation and physical limitations.

### Avoidant coping

Some participants focused on the negative elements of their diagnosis (limitations regarding work, physical, emotional aspects, etc.), which made it difficult for them to continue with their daily activities. Therefore, they avoided these tasks, which caused them to postpone coping at a certain point, which implied an immobilizing reflection on the fact, hiding the diagnosis from those close to them and seeking different medical opinions before starting any treatment.

*Well, to get closer, to verify, to have other opinions, until I rectified that, if it really was cancer, and to start with the treatment, right? TC, 56 years old.*

Participants also reported focusing on other thoughts and activities in order not to focus on the disease, thus seeking cognitive and behavioral distraction, including family activities, helping others and having behaviors related to the improvement of their health. It is relevant to point out that although these distractors reorganize the patients’ activities, they are also necessary to balance their daily tasks. This was more significant in stage III patients. 

*Yes, to avoid thinking about my illness, I look for more things to do, if I finish cooking, washing the dishes, sewing, I give myself more work to do. It helps me to clear my mind and not have negative thoughts. MR, 48 years old.*

In general, women who use avoidant coping are indifferent to the condition, so that, when they consider the negative elements, they prefer to postpone coping, either by avoiding prioritizing the diagnosis or by increasing the time dedicated to other activities in order to reduce the stress they are experiencing. In this way, thoughts related to worsening physical and emotional health will decrease.

## DISCUSSION

The psychological coping of the patients is presented by means of emotional, resolutive and avoidant coping strategies. This is in agreement with the theoretical approaches previously mentioned ^18^. However, in contrast to previous studies ^17^^,^^20^, which reported that resolute coping was the most used, our results showed that the emotional coping strategy was the most frequently used ^19^^,^^22^. This may be due to the pandemic context, in which people required greater social support from close relationships. Patients with chronic diseases were at greater risk of Covid-19, this produced intense fear, together with sleep problems, anxiety, and particularly the need for psychological help, which would facilitate communication with family and friends and allow them to watch over their own mental health ^32^.

Continuing with emotional coping, the need to feel trust and empathy, as well as being understood by people close to them, is evident ^18^^,^^33^. On the other hand, regarding religious coping, the patients strengthened their beliefs, which decreased the tension caused by the condition; this may be due to the fact that religion promotes feelings of faith, hope and strength, thus diminishing the perception of the diagnosis as a threat ^18^^,^^19^^,^^22^.

Next, in terms of focusing on positive consequences, participants engaged in new entertainment activities, which favors coping ^22^. This produced a positive reinterpretation of the situation, which contributed to a positive acceptance of the disease ^7^^,^^18^^,^^19^^,^^22^. This acceptance may be due to the fact that the more women are aware of their diagnosis, the more opportunities they will have to regain control of their lives ^19^. Therefore, it can be argued that women revalue their family, personal and religious life, which allows them to continue with their daily activities ^19^^,^^22^.

Regarding resolution coping, participants acted diligently in the face of their condition, which increases behaviors related to following medical indications, as well as positive emotions about their diagnosis ^17^^,^^18^^,^^20^. Participants followed the guidelines provided by the medical staff in a meticulous manner, improving their perception of control and decreasing the impression of threat ^17^^,^^19^^,^^20^.

Likewise, women seek professional help to cope with their condition, which agrees with previous research ^23^, this is due to the intervention methods used by the professionals to promote the balance of emotions ^33^^,^^34^. It should be noted that professional support is fundamental, therefore if it decreases, coping would be at risk ^23^^,^^33^. Along the same lines, women omit daily activities related to different aspects of their lives when carrying out actions to mitigate the situation ^22^.

On the other hand, regarding avoidant coping, participants initially focused on the negative elements of their illness, leading to emotions of distress that are expressed through crying, which, if not sustained over prolonged periods of time, can contribute to adaptation ^18^. Likewise, they postponed their coping strategies in order to adapt to their disease, therefore, they did not take into account the development of an additional plan due to the short time elapsed since the knowledge of their diagnosis ^18^^,^^19^. 

Furthermore, participants, mainly those in stage III, resorted to cognitive and behavioral distraction. In this sense, they carry out other activities, avoiding focusing on their diagnosis ^18^^,^^34^. Thus, patients seek balance between following medical advice and engaging in distracting activities ^7^.

One of the limitations of this study is that the interviews were conducted via telephone calls, which prevented the collection of nonverbal information. Other limitations include issues with the telephone signal, as well as not being able to ensure that the patient was in a suitable space to talk about sensitive issues, which could have affected the participant’s performance. The implications are based on understanding how psychological coping is employed by the women to deal with illness.

In conclusion, when faced with the psychosocial consequences of breast cancer, women employed different strategies. The emotional coping strategy stands out, in which they tried to increase positive emotions, either with the support of people close to them, religious support or by focusing on the positive consequences of the disease, as they all provide feelings of accompaniment, understanding, strength and tranquility. However, these strategies are also related to the personality of the patient, who, by having a more optimistic posture, try to face the condition similarly, avoiding focusing on the consequences and negative emotions. They also used problem-solving strategies, which helped them channel their efforts into taking action in the face of the disease, especially regarding medical care and treatment, therefore, they left aside other activities, postponing or skipping them in order to concentrate all their effort on physical and emotional recovery. On the other hand, women also used strategies to take their minds off the disease, as they focused on other activities, even initiating new projects that emerged as a way to detach themselves from their current concerns about their overall health.

Finally, we recommend that health professionals, family and friends should encourage an empathetic attitude towards the patient, since their actions influence the psychological coping process, favoring or harming the emotional health of women with breast cancer. Also, we suggest to further explore topics such as structured planning with self-initiative and ideas of abandonment of treatment, which turned out to be minor issues in this research, as they were found in a minimum number of cases.
