# Supplementary material for: Psychological coping in female breast cancer patients from a Metropolitan Lima hospital
Source: Rev Peru Med Exp Salud Publica. 2022 Dec 22;39(4):400–7. doi: 10.17843/rpmesp.2022.394.12322 (PMC11397672; doi:10.17843/rpmesp.2022.394.12322)
Supplement: Translation

# Translation: Afrontamiento psicológico en pacientes mujeres con cáncer de mama de un hospital de Lima Metropolitana

**Authors:** Fiorella D. Rivera-Cruzatt, Pierina P. Cubillas-Espinoza, Eli L. Malvaceda-Espinoza

**Affiliations:** 1 Universidad San Ignacio de Loyola, Lima, Perú.

**Keywords:** Estrategias de Afrontamiento, Paciente, Mujeres, Neoplasia de Mama, Investigación Cualitativa

## Abstract

**Objetivos.:**

Comprender el afrontamiento psicológico en pacientes mujeres con cáncer de mama de un hospital de Lima Metropolitana.

**Materiales y métodos.:**

La investigación siguió un enfoque cualitativo con un diseño de análisis temático reflexivo. Se entrevistó a 16 mujeres con cáncer de mama entre los 35 y 65 años. El análisis de datos se realizó con apoyo del software ATLAS.ti 22.

**Resultados.:**

Se presentaron tres estrategias de afrontamiento psicológico: *afrontamiento emocional*, el cual se encontró con mayor intensidad, y comprende el apoyo de personas significativas, el afrontamiento religioso y la concentración en las consecuencias positivas, generando una reinterpretación positiva y la aceptación progresiva de la enfermedad; el *afrontamiento resolutivo*, caracterizado por la acción diligente, con seguimiento a las indicaciones, y la búsqueda de apoyo profesional. Finalmente, el *afrontamiento evasivo*, se centró en los elementos negativos, el aplazamiento del afrontamiento y la distracción cognitiva y conductual, siendo este último de suma relevancia para equilibrar las actividades en la vida cotidiana de las pacientes.

**Conclusiones.:**

Las mujeres emplearon con mayor frecuencia estrategias del estilo emocional, ya que intentan incrementar las emociones positivas, acompañado del apoyo religioso y del entorno; además, utilizaron estrategias relacionadas al estilo resolutivo, puesto que centraron sus acciones para recibir la atención y tratamiento médico, dejando de lado otras actividades; pese a ello, emplearon estrategias para desenfocarse del padecimiento y así desligarse de sus preocupaciones.

## INTRODUCCIÓN

En el 2020, el cáncer de mama ocasionó la muerte de aproximadamente 685 000 mujeres a nivel mundial, asimismo, fue detectado en cerca de 2,2 millones de mujeres ^1^. En ese año, en el Perú se diagnosticaron 6860 casos, de esta cifra, el 63% tenían edades entre 35 y 64 años ^2^ ^,^ ^3^. El tratamiento para este padecimiento se ve afectado por el estado de salud, alteraciones en la alimentación, conductas de consumo y métodos anticonceptivos de uso oral ^4^. Es así como su tratamiento médico, puede ir desde una cirugía a una extirpación general del tejido mamario, acompañado de quimioterapia, radioterapia, terapia farmacológica, entre otros; estos tratamientos pueden desencadenar efectos secundarios tanto en el aspecto reproductivo y sexual, como la imposibilidad de tener hijos, lo cual genera mayores preocupaciones en las mujeres ^5^ ^,^ ^6^.

Asimismo, en el proceso de adaptación al diagnóstico, las mujeres enfrentan estados de vulnerabilidad, confusión, preocupación, desesperanza e incertidumbre ^7^ ^-^ ^10^. Ello genera una disminución de su autoestima ^11^, impresión de inferioridad ^7^, emociones de enojo, culpa, pena y miedo ^7^ ^,^ ^8^ ^,^ ^12^ ^,^ ^13^, incluso puede desencadenar trastornos como depresión y ansiedad ^7^ ^,^ ^14^, así como también elevados niveles de estrés ^7^ ^,^ ^9^ ^,^ ^10^. Dichos efectos psicológicos se incrementan en un contexto de pandemia, puesto que es más difícil obtener elementos esenciales al mantener el distanciamiento social, asimismo, las mujeres prefieren mantener comunicación con grupos de apoyo en línea, a raíz de que perciben un apoyo social limitado en amistades y familiares ^4^ ^,^ ^15^.

Mientras que, cuando se brinda apoyo familiar, la presencia de dificultades económicas y fallas en la comunicación provocan la separación de algunos integrantes de la familia, ocasionando sentimientos de frustración y malentendidos, incluso cuando dicho apoyo es positivo, se puede generar dependencia física o emocional ^12^ ^,^ ^13^. Por ello, es indispensable entender las estrategias de afrontamiento que las mujeres utilizan durante y después de conocer su diagnóstico ^7^ ^,^ ^14^. En ese sentido, el afrontamiento psicológico es entendido como las tareas cognitivas o conductuales realizadas por la persona, para lograr un mantenimiento de su adaptación psicosocial ante una problemática ^16^. Dicho afrontamiento se divide en estrategias, con base a un grupo de acciones que varían frente al ambiente de la persona y otros factores ^17^. 

Al respecto, *el estilo de afrontamiento emocional* ^16^ ^,^ ^18^ comprende las estrategias (apoyo familiar y amical, apoyo religioso, transformación positiva, negación y aceptación) que permiten ajustar el nivel de sufrimiento emocional que genera dicha condición de salud ^19^. Además, *el estilo de afrontamiento resolutivo* ^16^ ^,^ ^18^, contiene estrategias (diligencia, apoyo profesional, aplazamiento de acciones afrontativas, planeamiento, omisión de actividades ordinarias), con el objetivo de disminuir el estrés provocado, y promover actitudes psicológicas y emocionales positivas ^17^ ^,^ ^18^ ^,^ ^20^. También, el *estilo de afrontamiento evitativo* ^18^, que incluye estrategias (concentración y manifestación de los efectos adversos, distracción cognitiva y distracción conductual), donde el individuo demuestra desinterés frente al acontecimiento intimidante ^18^ ^,^ ^19^ con el fin de evitar la problemática.

Respecto a las investigaciones precedentes, las estrategias de afrontamiento más utilizadas por las mujeres a nivel global están relacionadas a resolver el conflicto ^17^ ^,^ ^20^. Además, variables como la edad, el estado sentimental y la escolaridad son tomadas en cuenta al momento de optar por el empleo de una estrategia u otra ^21^. Mientras en el Perú, el estilo de afrontamiento más utilizado es el emocional, que implica las estrategias: afrontamiento religioso y reinterpretación positiva ^19^ ^,^ ^22^; no obstante, aquellas estrategias del estilo evitativo son menos frecuentes ^22^. Frente a ello, las pacientes demuestran que, mientras más apoyo profesional reciban, presentarán un mayor afrontamiento psicológico frente al diagnóstico ^7^; asimismo, dicho apoyo puede provenir del personal de salud encargado de su atención, mediante un trato cálido y empático, impulsando hábitos alimentarios, de higiene y para demostrar un estado emocional positivo ^23^. 

Sin embargo, aunque estas investigaciones fueron realizadas en Perú, han seguido un enfoque cuantitativo, donde no se recabó información del fenómeno a profundidad, por ello se busca atender dicho vacío teórico a través de la evidencia empírica mediante herramientas metodológicas cualitativas. A su vez, servirá para que más mujeres puedan emplear un afrontamiento más relacionado a un ambiente positivo y generar mayor importancia sobre el apoyo psicológico que se les brinda. De acuerdo con lo señalado, se plantea el objetivo de comprender el afrontamiento psicológico en pacientes mujeres con cáncer de mama de un hospital de Lima Metropolitana.

**MENSAJES CLAVE**

Motivación para realizar el estudio: es importante conocer el empleo de estrategias de afrontamiento que utilizan las mujeres que tienen cáncer de mama, con la finalidad de contribuir a su bienestar físico y emocional.

Principales hallazgos: las estrategias relacionadas al ámbito emocional son utilizadas con mayor intensidad, asimismo, conllevan a una aceptación progresiva de la enfermedad. La distracción cognitiva y conductual resultan necesarias para equilibrar las actividades cotidianas de las pacientes.

Implicancias: el comprender cómo las mujeres afrontan su enfermedad, permite la generación de estrategias desde la atención primaria para la mejora de su bienestar.

## MATERIALES Y MÉTODOS

### Tipo y diseño

La investigación siguió un enfoque cualitativo y un diseño de análisis temático reflexivo, el primero permite indagar a profundidad el tema de estudio en su ambiente natural, analizando dichos datos y determinando categorías de este ^24^, mientras el diseño posibilita comprender la información obtenida, de manera que se enfoca en los temas determinados por los participantes y explicados por los investigadores, asociando los datos según patrones en temáticas relevantes ^25^.

### Participantes

Participaron 16 mujeres, con edades desde los 35 a 65 años (tabla 1), que presentan cáncer de mama y que reciben asistencia médica en un hospital de Lima Metropolitana, el cual brinda servicio de manera pública de nivel de atención 3 y especializado en pacientes oncológicas. La estrategia de muestreo fue por conveniencia, ello es utilizado cuando existe poca accesibilidad a la población ^26^, como en el presente estudio, donde la temática se asocia a padecimientos severos. Se incluyó a pacientes entre los estadios II y III, detectado como mínimo un año antes de esta investigación. Se excluyó a aquellas que presentaron comorbilidad física o alguna alteración psicopatológica, debido a que se manifiestan diferencias en cuanto al tratamiento, consecuencias físicas y psicológicas, proceso de adaptación al diagnóstico, apoyo profesional y familiar frente a sus necesidades, así como en la atención que brindan los hospitales que se especializan en el cáncer ^5^ ^,^ ^13^.

**Tabla 1:** Características de las participantes.

Seudónimo	Edad	Ocupación	Estado civil	Composición familiar	Estadio del diagnóstico	Edad de diagnóstico
AA	64	Enfermera	Casada	Esposo e hijo	II	63
OS	46	RdH	Casada	Esposo e hijos	II	44
DA	59	RdH	Casada	Esposo e hijo	III	54
DN	52	Téc. enfermería	Casada	Esposo e hijos	II	51
JI	59	Independiente	Casada	Hijos y sobrina	II	58
TC	56	Administradora	Casada	Esposo e hijos	II	55
MR	48	Profesora	Casada	Esposo e hijos	II	47
AR	65	RdH	Soltera	Hermana y sobrina	III	60
SU	51	Independiente	Soltera	Madre y hermanos	III	49
YJ	59	RdH	Casada	Esposo e hijos	III	56
LG	64	Independiente	Casada	Esposo	III	63
TH	59	RdH	Casada	Esposo e hijas	III	58
SF	51	Cosmetóloga	Casada	Esposo	II	48
RS	48	Independiente	Casada	Hijo, padres y hermanos	III	43
SN	35	Contadora	Soltera	Padre	III	31
CR	51	RdH	Casada	Esposo e hijos	II	46

RdH: responsables del hogar, II: estadio 2 del cáncer de mama, III: estadio 3 del cáncer de mama.

### Instrumentos de recolección de información

Se empleó una entrevista semiestructurada (tabla 2), construida con base en los aportes teóricos acerca del afrontamiento psicológico explicado desde los tópicos de afrontamiento emocional, resolutivo y evasivo ^18^. La guía de entrevista fue auditada por diez jueces expertos, siendo profesionales de la salud con conocimiento en la población de pacientes con cáncer, quienes evaluaron la claridad, coherencia y representatividad de las preguntas, frente a lo cual se obtuvo aprobación para iniciar con las mismas; asimismo, se realizó una entrevista piloto, mediante llamada telefónica, a partir de lo cual se pudo afinar las preguntas para la guía de entrevista final.

**Tabla 2:** Preguntas orientadoras.

Tópicos	Preguntas orientadoras
Afrontamiento emocional	¿A quiénes acudió para que la apoyaran emocionalmente en el proceso de su padecimiento? ¿Por qué buscar ese apoyo en otras personas?
Afrontamiento resolutivo	¿En qué momento Ud. comenzó a actuar para afrontar dicho padecimiento? ¿Considera que sus acciones se dieron en el momento oportuno? ¿Por qué?
Afrontamiento evasivo	¿En qué aspectos de su padecimiento considera que se centra Ud.? ¿De qué manera expresa las emociones obtenidas a raíz de estas consecuencias? ¿Lo considera adecuado?

Fuente: elaboración propia

### Procedimientos

Previamente, mediante una red social, se entabló comunicación con la presidenta de un grupo de pacientes con cáncer de mama de un hospital en Lima, frente a lo cual, se recibió su aprobación para contactar por primera vez a dichas pacientes. En ese sentido, se escribió a 37 mujeres, de ellas 17 estuvieron de acuerdo con participar en la investigación; sin embargo, solo 16 cumplieron con los criterios de inclusión. Posteriormente, se les indicó el objetivo y aceptaron el consentimiento informado de manera verbal, del cual se grabaron audios. Asimismo, se indicó la confidencialidad, anonimato (uso de seudónimos), beneficencia y no maleficencia del estudio.

Posteriormente, se acordó la fecha para las entrevistas, que se realizaron a través de llamadas telefónicas, donde se procuró que las personas se encuentren solas y en un espacio cómodo (habitualmente, la sala de su casa). Las entrevistas se realizaron entre septiembre y octubre del 2020, y tuvieron una duración promedio de 40 minutos. La realización y transcripción literal de las entrevistas fueron desarrolladas por las investigadoras FR y PC, quienes se dividieron el total de entrevistas equitativamente. No hubo personas que se negaron a participar o abandonaron el estudio y no se otorgó pago alguno por la participación en las entrevistas, siendo realizadas en una sesión (no hubo repeticiones) y en español.

### Análisis de datos

Se siguieron las pautas del análisis temático reflexivo con un método de corte híbrido ^25^. Se inició con la *familiarización con los datos*, mediante las transcripciones. Posteriormente, se generaron *códigos iniciales* adscritos a las citas relevantes del documento, en este punto se realizó un doble ciclo de codificación por patrones, axial y selectiva ^27^; en paralelo, se realizó la *generación, revisión y definición de temas*, que fueron identificados mediante el método comparativo constante, agrupándose los códigos en categorías. Por último, se desarrolló el *reporte de resultados*, donde se encontraron las tácticas de generación de significado ^28^, tales como la *identificación de patrones*, que permite evidenciar la saturación de los códigos y/o de categorías; el *conteo*, que permite manifestar la magnitud de los códigos a lo largo del análisis; y la *densidad*, la cual evidencia el poder explicativo de los códigos, a través de sus conexiones con otros códigos y/o categorías. Se consideró como temas mayores aquellas categorías que hayan cumplido al menos una de las tácticas antes señaladas. El análisis de fue realizado principalmente por FR y PC, y discutieron y absolvieron junto a EM las discrepancias en torno a la codificación. El análisis se realizó con el apoyo del software ATLAS.ti 22.

### Criterios éticos y de calidad

Se obtuvo el permiso del comité de ética de la escuela de Posgrado de la Universidad Peruana Unión (Nº 2022-CE-EPG-0000116). Este artículo se adapta a los criterios del Consolidated Criteria for Reporting Qualitative Research (COREQ) ^29^. 

También, cumple con criterios de calidad tales como *credibilidad* ^(^ ^30^, debido a que se realizó la entrega de las transcripciones y resultados del estudio a las participantes para verificar la información y obtener sus apreciaciones; sin embargo, no hubo comentarios adicionales por parte de ellas. Asimismo, cumple con el criterio de *confirmabilidad* ^30^ *, *puesto que tanto la recolección de datos, el análisis y los resultados pueden rastrearse y verificarse hasta su origen, lo cual genera una cadena lógica de evidencia. Seguidamente, la *auditabilidad* ^31^, que implicó la triangulación de investigadores al auditar la guía de entrevista mediante 10 jueces expertos.

## RESULTADOS

Las 16 participantes entrevistadas tenían una edad promedio de 54 años. Respecto al estadio, se encontraron equitativamente distribuidas entre los estadios II y III. En cuanto a sus actividades, seis indicaron ser responsables del hogar, mientras las otras 10 ejercían un oficio o profesión. La mayoría de las mujeres estaban casadas, su estructura familiar estaba comprendida por el cónyuge e hijos o uno de ellos. Las edades en que fueron diagnosticadas varían entre los 31 y 63 años. 

De acuerdo con el análisis, el cáncer de mama genera distintas* consecuencias psicosociales* entre las cuales se evidencian la dependencia, la debilidad física, así como sentimientos de tristeza, depresión y frustración. 

*Ahí yo estaba con más dolor del cuerpo, me dolían los huesos, ya no podía caminar tanto y estaba en silla de ruedas. SN, 35 años.*

A pesar de ello, las mujeres presentan *deseos de continuar el tratamiento*. Lo anterior conduce a las participantes a afrontar psicológicamente su enfermedad a través de distintas estrategias tales como el afrontamiento emocional, resolutivo y evitativo, lo cual se pasará a abordar a continuación (figura 1 ).

**Figura 1:** Mapa semántico en torno a las estrategias de afrontamiento en pacientes mujeres con cáncer de mama.

### Afrontamiento emocional

Esta estrategia de afrontamiento fue la señalada con mayor intensidad e implica las acciones centradas en las emociones, con la finalidad de mejorar su situación actual. Al respecto, las pacientes buscaron *apoyo social en las personas significativas*, sea de familiares, amistades, grupos de apoyo y religiosos, siendo ello de relevancia en las pacientes del estadio III. 

*En mi caso, mis amigas de trabajo me dijeron: anímate, SN, busca otra opinión*. SN, 35 años. 

Esta es una forma directa de hacer frente a la situación, con la finalidad de *buscar comprensión, confianza y empatía*. Asimismo, presentaron *afrontamiento religioso*, donde se aproximaron más y fortalecieron sus creencias. 

*Soy una persona creyente, ese fue mi soporte […] yo creía que la voluntad de Dios estaba siendo clara con las recaídas y tenía que aceptarlo […] estoy dando para adelante para mi sanación. *CR, 51 años. 

Por otra parte, también evidenciaron el *concentrarse en las consecuencias positivas* de la enfermedad, lo cual conlleva a que ellas generen una *reinterpretación positiva*, al reconocen experiencias beneficiosas frente a lo vivido; en este punto, señalan el realizar actividades nuevas y revalorar las actividades cotidianas (pasar tiempo en familia y mejora del autocuidado).

*Pero a raíz de este diagnóstico, he decidido hacer cosas que siempre estuve pensando en hacerlas más adelante. Entonces, ahora me he dedicado a la música, pienso enseñar, eso me llena el alma, la vida y todo. Es algo que me divierte. *TC, 56 años.

Un elemento que se genera a partir de la reinterpretación positiva es la *aceptación progresiva de la enfermedad,* así, si bien en un inicio presentaron sentimientos de miedo y preocupación, posteriormente experimentaron sorpresa y tranquilidad, llegando a aceptar la enfermedad.

*Ya uno tiene que convivir con eso, ya no queda de otra, ya, aunque aún extrañas tu vida antes, las cosas que hacías. *JN, 58 años.

En ese sentido, las mujeres buscaron el apoyo de su entorno cercano, ello en búsqueda de comprensión, confianza y empatía, lo cual las hace sentirse acompañadas para lograr controlar las emociones ocasionadas por el padecimiento. Asimismo, acercarse a la religión, hizo que sientan que el padecimiento es un obstáculo que superar, por lo que ello les brindó fe y fortaleza, logrando sentirse reconfortadas; mientras se concentraron en las consecuencias positivas, ya sea para evitar incrementar emociones negativas o porque tienen una perspectiva más optimista de las situaciones de la vida acorde con su personalidad, de manera que generaron la reinterpretación positiva, logrando la aceptación progresiva de la enfermedad. 

### Afrontamiento resolutivo

Las pacientes, mediante el transcurso de sus primeras experiencias con el diagnóstico, realizaron acciones *diligentes* para intervenir directamente ante las sospechas de su dolencia, especialmente al buscar la opinión médica para confirmar su diagnóstico y conocer sobre los posibles tratamientos que tendría que llevar. Frente a ello, se enfocaron en el *seguimiento de indicaciones médicas*. 

*Simplemente seguir lo que me decían los doctores ¿No? Ellos son los que te dicen, primero la operación, la mastectomía, radiación, biopsia, una cita para esto, para lo otro, son ellos a los que uno tiene que seguir*. SF, 51 años. 

De modo que, las pacientes realizaron la *búsqueda de apoyo profesional, *ya sea en cuanto al área física y psicológica, con la finalidad de disminuir los efectos adversos del padecimiento, siendo ello representativo en mujeres del estadio II. 

*Cuando yo fui donde el psicólogo, me ayudó muchísimo porque sentía que esa persona me hacía sentir más tranquila, sentí sus abrazos de la doctora que me apoyó, siempre me dice: ‘Estás superándolo, estás bien.’ *DN, 52 años. 

Por otro lado, indicaron *omitir actividades cotidianas, *sean laborales, académicas y del hogar que realizaban antes de enterarse de su diagnóstico, con la finalidad de centrar todos sus esfuerzos temporales, mentales y físicos para llevar a cabo el tratamiento y sus implicancias.

*Hasta antes era todo normal, pero al momento de enterarme, a partir de ello, ya estoy casi un año sin trabajar, puesto de que el tratamiento, las operaciones, la quimio y ahora la radio me toman demasiado tiempo […] entonces, es prácticamente dedicarle todo ese tiempo a solo curarte. *TC, 56 años.

De manera que, ante las sospechas del cáncer de mama, se genera una incertidumbre, por lo que, para disminuirla, las pacientes buscan atención médica para conocer su padecimiento, mientras cuando ya lo conocen, se enfocan en cumplir con las indicaciones médicas, ello debido a la toma de decisiones apresuradas para evitar el agravamiento de la salud. Es así que, buscan apoyo profesional para mejorar su condición física y mental; así también, se ven comprometidas a dejar de lado actividades rutinarias, estudios y trabajo, por la falta de tiempo, motivación e impedimentos físicos. 

### Afrontamiento evasivo

Con relación a esta estrategia, las mujeres se *centraron en los elementos negativos* de su diagnóstico (limitaciones en el ámbito laboral, físico, emocional, etc.) lo cual les dificulta continuar con sus actividades cotidianas, frente a lo cual omitieron dichas labores, ello ocasionó que en cierto momento, *aplazaran el afrontamiento*, lo cual implicó la reflexión inmovilizante ante el hecho, el ocultamiento del diagnóstico a las personas cercanas y la búsqueda de diferentes opiniones médicas antes de iniciar con algún tratamiento. 

*Bueno, acercarme, verificar, tener otras opiniones, hasta llegar a rectificar que, si realmente era cáncer, y ya empezar con el tratamiento ¿No?* TC, 56 años.

Asimismo, señalan centrarse en otros pensamientos y actividades con el fin de no enfocarse en su enfermedad, de tal manera, buscan la *distracción cognitiva y conductual*, que incluye la realización de actividades familiares, quehaceres de ayuda a otros y conductas relacionadas a la mejora de otros aspectos de la salud. Resulta relevante indicar que dichos distractores si bien descentran las actividades de las pacientes, son también necesarios para equilibrar sus actividades cotidianas. Ello resultó más significativo en pacientes del estadio III. 

*Sí, para evitar pensar en mi enfermedad, busco más cosas para hacer, si terminé de cocinar, lavo los platos, coser, yo misma me doy más labor. Me ayuda a despejar la mente y no tener pensamientos negativos*. MR, 48 años. 

En general, en el afrontamiento evasivo, las mujeres se muestran indiferentes con el padecimiento, de modo que, cuando consideran los elementos negativos, prefieren aplazar el afrontamiento, ya sea evitando priorizar el diagnóstico en su vida o incrementando la realización de otras actividades para minorar la situación estresante que vienen atravesando. Es así como, disminuirá los pensamientos relacionados al agravamiento de la salud física y emocional.

## DISCUSIÓN

El afrontamiento psicológico de las pacientes se presenta mediante las estrategias de afrontamiento emocional, resolutivo y evasivo. Ello concuerda con los planteamientos teóricos indicados anteriormente ^18^. Sin embargo, a diferencia de estudios previos ^17^ ^,^ ^20^, quienes indicaron que el afrontamiento resolutivo era el más utilizado, en este estudio resaltó el afrontamiento emocional ^19^ ^,^ ^22^. Ello se puede deber al contexto de pandemia, donde se requirió de un mayor apoyo social por parte de las personas significativas, puesto que, aquellas pacientes con enfermedades crónicas se encontraban en la población de riesgo a causa del Covid-19, lo que generó un miedo intenso, aunado a los problemas para dormir, ansiedad, sobre todo la necesidad de ayuda psicológica, permanecer comunicado con familiares y amistades, así como velar por su salud mental ^32^.

Siguiendo con el afrontamiento emocional, se evidencia la necesidad de sentir comprensión, confianza y empatía en personas cercanas ^18^ ^,^ ^33^. Por otro lado, con relación al afrontamiento religioso, las pacientes fortalecieron sus creencias, produciendo una disminución de la tensión originada por el padecimiento; esto puede ser ocasionado a raíz de que la religión promueve sentimientos de fe, esperanza y fortaleza, con lo cual disminuye la percepción del diagnóstico como una amenaza ^18^ ^,^ ^19^ ^,^ ^22^.

Seguidamente, en cuanto a la concentración en las consecuencias positivas, se realizaron actividades novedosas de entretenimiento, favoreciendo el afrontamiento ^22^. Esta concentración produjo la reinterpretación positiva de la situación, lo cual contribuyó a la aceptación positiva de la enfermedad ^7^ ^,^ ^18^ ^,^ ^19^ ^,^ ^22^. Esta aceptación puede deberse a que mientras más conocimiento tengan las mujeres de su diagnóstico, tendrán mayores oportunidades para retomar el control de su vida ^19^. En relación con lo anterior, se puede afirmar que las mujeres revaloran su vida familiar, personal y religiosa, lo cual les permite continuar con sus actividades diarias ^19^ ^,^ ^22^. 

Respecto al afrontamiento resolutivo, las participantes actuaron diligentemente ante su padecimiento, ello incrementa el comportamiento de seguimiento de indicaciones médicas, así como las emociones positivas sobre su diagnóstico ^17^ ^,^ ^18^ ^,^ ^20^. Al respecto, las mujeres continuaron las pautas brindadas por el personal médico de forma minuciosa, generando un mayor nivel de percepción de control y disminuyendo la impresión de amenaza ^17^ ^,^ ^19^ ^,^ ^20^. 

Asimismo, las mujeres realizan la búsqueda de ayuda profesional para afrontar su situación, ello concuerda con otras investigaciones ^23^, esto se debería a los métodos de intervención utilizados por dichos profesionales para impulsar el equilibrio de las emociones ^33^ ^,^ ^34^. Cabe rescatar que el apoyo profesional es fundamental, pues si este disminuye, se encontraría en riesgo el afrontamiento ^23^ ^,^ ^33^. En esta misma línea se observa, que las mujeres al realizar acciones para mitigar su situación, omiten actividades cotidianas vinculadas a diferentes aspectos de su vida ^22^.

Por otra parte, en cuanto al afrontamiento evasivo, las mujeres inicialmente se centraron en los elementos negativos de sus enfermedades, las cuales generan el surgimiento de emociones de angustia que es expresado mediante el llanto, el cual, si no se expande a periodos prolongados, puede contribuir a la adaptación ^18^. Asimismo, aplazaron sus acciones de afrontamiento para adaptarse a su enfermedad, en tal sentido, no tomaron en cuenta el desarrollar un plan adicional por el corto tiempo transcurrido desde el conocimiento de su diagnóstico ^18^ ^,^ ^19^.

Continuando con lo anterior, las pacientes, principalmente las del estadio III, recurren a la distracción cognitiva y conductual. En ese sentido realizan otras actividades, evitando la focalización en su diagnóstico ^18^ ^,^ ^34^. De esa manera, las pacientes buscan un equilibrio entre seguir indicaciones médicas a favor de su diagnóstico y realizar actividades que les generen distracción ^7^.

Como limitaciones se debe mencionar que las entrevistas fueron aplicadas a través de llamadas telefónicas, ello impidió el recojo de información no verbal, se presentó a su vez dificultades con la señal telefónica, asimismo, el asegurar que la paciente esté en un espacio adecuado dentro del hogar para conversar sobre temas sensibles, ello pudo afectar el desenvolvimiento de las participantes. Las implicancias se basan en comprender cómo es empleado el afrontamiento psicológico por las mujeres para afrontar su enfermedad. 

En conclusión, ante las consecuencias psicosociales del cáncer de mama, las mujeres emplearon distintas estrategias, de modo que, resalta el estilo emocional, debido a que intentan incrementar las emociones positivas, ya sea con el apoyo del entorno cercano, apoyo religioso o centrándose en las consecuencias positivas del padecimiento, ya que todas generan una sensación de acompañamiento, comprensión, fortaleza y tranquilidad; sin embargo, dichas estrategias están también relacionadas a la propia personalidad de la paciente, quienes al tener una postura más optimista, procuran afrontar su padecimiento de la misma manera, evitando centrarse tanto en las consecuencias como emociones negativas. También, utilizan estrategias del estilo resolutivo, es así como centran sus acciones en actuar frente al padecimiento, especialmente relacionadas a la atención y tratamiento médico, de manera que dejan de lado otras actividades, aplazando u omitiéndolas para poder focalizar todos sus esfuerzos en recuperarse a nivel físico y emocional. Por otra parte, las mujeres utilizan estrategias para desenfocarse del padecimiento, ya que se centran en otras actividades, incluso iniciando nuevos proyectos que surgen como una forma para desligarse de sus actuales preocupaciones en torno a su salud integral.

Finalmente, se recomienda que desde los hospitales o clínicas tanto profesionales de la salud como familiares y amistades promuevan un trato empático hacia la paciente, puesto que sus acciones influencian el afrontamiento psicológico, favoreciendo o perjudicando la salud emocional de las mujeres con cáncer de mama. Así también, se sugiere profundizar sobre temas como la planificación estructurada con iniciativa propia y las ideas de abandono del tratamiento, los cuales resultaron ser temas menores de la investigación, al presentarse en una mínima cantidad de testimonios.
